# Dissociation process of polyalanine aggregates by free electron laser irradiation

**DOI:** 10.1371/journal.pone.0291093

**Published:** 2023-09-08

**Authors:** Hisashi Okumura, Satoru G. Itoh, Heishun Zen, Kazuhiro Nakamura

**Affiliations:** 1 Exploratory Research Center on Life and Living Systems (ExCELLS), National Institutes of Natural Sciences, Okazaki, Aichi, Japan; 2 Institute for Molecular Science, National Institutes of Natural Sciences, Okazaki, Aichi, Japan; 3 Graduate Institute for Advanced Studies, SOKENDAI, Okazaki, Aichi, Japan; 4 Institute of Advanced Energy, Kyoto University, Gokasho, Uji, Kyoto, Japan; 5 Department of Laboratory Sciences, Gunma University Graduate School of Health Sciences, Maebashi, Gunma, Japan; University of Helsinki, FINLAND

## Abstract

Polyalanine (polyA) disease-causative proteins with an expansion of alanine repeats can be aggregated. Although curative treatments for polyA diseases have not been explored, the dissociation of polyA aggregates likely reduces the cytotoxicity of polyA. Mid-infrared free electron laser (FEL) successfully dissociated multiple aggregates. However, whether the FEL dissociates polyA aggregates like other aggregates has not been tested. Here, we show that FEL at 6.1 μm experimentally weakened the extent of aggregation of a peptide with 13 alanine repeats (13A), and the irradiated 13A exerted lesser cytotoxicity to neuron-like cells than non-irradiated 13A. Then, we applied molecular dynamics (MD) simulation to follow the dissociation process by FEL. We successfully observed how the intermolecular β-sheet of polyA aggregates was dissociated and separated into monomers with helix structures upon FEL irradiation. After the dissociation by FEL, water molecules inhibited the reformation of polyA aggregates. We recently verified the same dissociation process using FEL-treated amyloid-β aggregates. Thus, a common mechanism underlies the dissociation of different protein aggregates that cause different diseases, polyA disease and Alzheimer’s disease. However, MD simulation indicated that polyA aggregates are less easily dissociated than amyloid-β aggregates and require longer laser irradiation due to hydrophobic alanine repeats.

## 1 Introduction

Polyalanine (polyA) disease is a class of triplet expansion diseases. The trinucleotide repeats in polyA disease-causative genes are expanded in the patients, and the resultant proteins with expanded polyA are the cause of the disease. Since the causative proteins include transcription factors that play crucial roles in the development of multiple organs, most polyA diseases accompany congenital defects in multiple types of cells. In general, both the loss of normal function and a toxic gain-of-function contribute to the pathogenesis of neurodegenerative disorders. As indirect evidence of loss of normal function in polyA diseases, the amount of polyA disease-causative proteins was reduced in mice with a polyalanine expansion mutation [1]. The reduced protein level can likely contribute to the dysfunction of cells because the mice with expanded polyA repeat in a protein showed the same phenotypes as those seen in other mutant mice with reduced levels of the same protein [2].

In addition, toxic gain-of-function by the presence of expanded polyA-containing proteins might contribute to the pathogenesis because the expansions often lead to unusual aggregation of the proteins. For instance, nuclear aggregates containing polyadenylate-binding nuclear protein 1 (PABPN1), the responsible molecule of oculopharyngeal muscular dystrophy, were detected in the patients [3, 4]. Likewise, intranuclear aggregation and apoptosis were found in the skeletal muscle of the transgenic mice with expanded polyA-containing PABPN1 [5–8]. In addition to the presence of polyA aggregates, the aggregates functionally induce cytotoxicity because aggregated polyA peptide with 13 alanine repeats (13A) was aversive to cultured cells and the mouse brain [9]. Therefore, the elimination of polyA aggregates is expected to reduce polyA cytotoxicity.

Irradiation of mid-infrared free electron laser (FEL) tuned at 6.0–6.2 μm dissociated multiple types of aggregates [10–14] due to vibrational excitation at amide I band (C = O stretch). Thus, applying FEL might offer an opportunity to explore therapeutic approaches for polyA diseases.

Molecular dynamics (MD) simulation is a useful computational research tool to reveal the conformational change and dynamics of protein aggregates [15–25]. Atomic and molecular motions are calculated based on Newton’s equations of motion on a computer. In particular, nonequilibrium MD simulation is used to understand the molecular-level mechanism of protein aggregate dissociation by FEL [26, 27]. In the process of FEL irradiation, MD simulation is performed under a time-dependent electric field that mimics FEL. Such simulations have been performed for amyloid-β (Aβ) peptide aggregates and revealed that FEL at 6.1 μm successfully dissociated Aβ fibrils [26, 27]. The simulation also verified that water molecules interfere with the reformation of the β-sheet structure. However, it is not known whether FEL at 6.1 μm dissociates polyA aggregates, and if so, water molecules are involved in the dissociation. In this study, we conducted a wet experiment in which polyA aggregates were irradiated with FEL. Then, we performed nonequilibrium MD simulations of polyA aggregates with FEL irradiation.

## 2 Materials and methods

### 2.1 FEL irradiation experiment

#### 2.1.1 Polyalanine peptide

A TAMRA-labeled peptide containing 13 uninterrupted alanine repeats (13A) with a purity of more than 95% was Synthesized (GL Biochem, Shanghai, China). The sequence is 5-TAMRA-KKWAAAAAAAAAAAAAKK-NH_2_. The sequence was the same as in previous literature [28]. The concentration of the stock solution of 13A was 10 mg/ml in a 1:1 mixture of trifluoroacetic acid and hexafluoroisopropanol, as described [9, 29, 30].

#### 2.1.2 FEL irradiation

FEL pulses in Kyoto University Free Electron Laser (KU-FEL) facility [31] consist of macro- and micro-pulses. The macro-pulse length (*T*_*macro*_) is approximately 2-μs with the micro-pulse repetition rate (*f*_*micro*_) of 2856 MHz. It means that the micro-pulses are irradiated every 350 ps. The typical micro-pulse length (*T*_*micro*_) is shorter than 1 ps. The FEL beam at 6.1 μm was focused at the center of the 13A aggregates solution (1 mg/ml in water) with the spot size of 0.2 cm. The irradiation was applied for 10 min at room temperature. The macro-pulse repetition rate *f*_*macro*_ and the macro-pulse energy of the FEL beam were 1.76 Hz and approximately 20 mJ, respectively. This energy corresponds to the maximum electric field of approximately 0.4×10^6^ V/cm. Because the temperature of the entire system hardly increased during the FEL irradiation experiment except for the potential momentary increase, we did not need to take steps to prevent temperature rise, such as adding water regularly.

#### 2.1.3 Dot blot analysis

TAMRA-labeled 13A in a solution containing 0.2% Triton X-100 and 25 mM EDTA was centrifuged at 22,000 g at 4°C for 30 min as essentially described [32]. The resultant supernatant was collected as the soluble fraction. Then, the pellet was re-dissolved with a same volume of a solution containing 2% SDS and 25 mM EDTA as the insoluble fraction.

One microliter of each fraction was dropped onto PVDF membrane pre-soaked with methanol. The TAMRA signal was detected by ImageQuant^TM^ LAS 4010 (GE Healthcare, Chalfont Saint Giles, UK). Mean signal intensity and the area of the signal were measured using Image J software and were multiplied to yield the amount of 13A in each fraction. Percentages of the amounts of 13A in insoluble fractions relative to the amounts in soluble fractions were calculated.

#### 2.1.4 PC12 cell culture

PC12 cells were purchased from RIKEN BRC and were cultured as described in 5% CO2 at 37°C [9, 29, 30]. 3 x 10^3^ cells in DMEM containing 10% FBS and antibiotics were plated in 96-well plates without coating with laminin. After the addition of polyA peptides to the culture medium at a concentration of 10 μg/ml, differentiation was induced by replacing the medium with DMEM containing 1% FBS, 0.25% BSA, and 50 ng/mL NGF. Then, the cells were cultured for 5 days.

For immunostaining, PC12 cells were fixed with 4% paraformaldehyde and then were incubated with anti-tyrosine hydroxylase antibody (Merck Millipore, Burlington, MA). Then, the cells were incubated with Alexa Fluor 488-labeled secondary antibody. Images were taken using a BZ9000 microscope (Keyence, Osaka, Japan). The length of each neurite of PC12 cells was measured using Image J software, and the total length of neurites per cell was calculated.

#### 2.1.5 Statistical analysis

Values were expressed as the mean, and error bars represented standard errors in the graph. Statistical significance was analyzed using ANOVA followed by Tukey test. When normality was not met, Kruskal-Wallis followed by Steel-Dwass analysis was used. Statistical significance was set at p < 0.05.

### 2.2 MD simulation

#### 2.2.1 Equilibrium MD simulation before nonequilibrium MD simulation

The initial structure of the amyloid fibril of polyA was modeled in the following procedures. Each polyA peptide consisted of 13 alanine residues, and the N-termini and the C-termini were blocked by the acetyl group and the N-methyl group, respectively. The model polyA sequence used here consisted of 13 alanine repeats without the flanking sequences (KKW and KK) at both sides. Because circular dichroism spectroscopy experiments have shown that polyA amyloid fibrils have an antiparallel β-sheet structure [9], we first performed equilibrium MD simulations of polyA amyloid fibrils with a single-layer antiparallel β-sheet structure. Unexpectedly this single-layer β-sheet structure separated into two β-sheets, and they were stacked and stabilized, as shown in S1 Movie. We thus created an initial structure model with two-layer antiparallel β-sheets to perform the following equilibrium and nonequilibrium MD simulations. One polyA amyloid fibril and 9,662 water molecules were placed in a cubic simulation box with a side length of 68.359 Å. The total number of atoms was 30,690. Six MD simulations were performed from different initial velocities for the statistical analysis.

**Fig 1:**
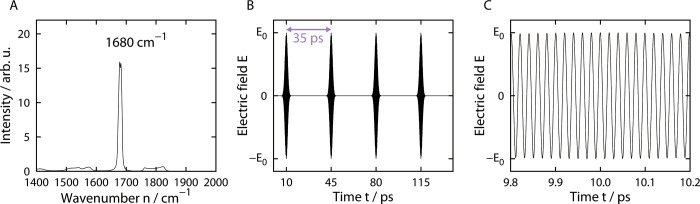
Determination of electric field that expresses FEL irradiation in MD simulation. (A) Infrared absorption spectrum of the backbone C = O stretching vibration. The resonance wavenumber was determined as 1680 cm^-1^. (B) Electric field pulses applied in the nonequilibrium MD simulations. (C) Enlarged view of the electric field in panel (B).

An infrared laser that corresponds to the backbone C = O stretching vibration (amide I band) is used in the IR-FEL experiments. We first performed equilibrium MD simulations to determine the C = O stretching mode of this model amyloid-fibril. The Generalized-Ensemble Molecular Biophysics (GEMB) program was used, which was developed by one of the authors (H. Okumura). This program has been applied to several protein and peptide systems [33–42]. We applied the AMBER ff14SB force field [43] to the polyA peptides. We used the TIP3P rigid body model [44] for the water by adopting the symplectic [45] quaternion scheme [46, 47]. The electrostatic potential was calculated using the particle mesh Ewald (PME) method [48]. The cut-off distance was 12.0 Å for the Lennard–Jones (LJ) potential. Reversible multiple time-step MD techniques were applied [49]. The time step was taken to be *Δt* = 0.5 fs for the bonding interactions of the peptide atoms, *Δt* = 2.0 fs for the LJ interactions, the real part of the PME calculation of the peptide atoms, and those between the peptide atoms and solvent molecules, and *Δt* = 4.0 fs for the LJ interaction, the real part of the PME calculation between the solvent molecules, and the reciprocal part of the PME calculation of all the atoms. Because the symplectic rigid body algorithm was used for the water molecules, *Δt* can be taken as long as 4.0 fs [47]. MD simulations were performed in the isothermal–isobaric ensemble for 50 ns from each initial condition. The temperature was controlled at 310 K by the Nosé–Hoover thermostat [50–52]. The pressure was controlled at 0.1 MPa using the Andersen barostat [53]. The first 10 ns of the simulations were regarded as the equilibration, and the following 40 ns were used to calculate the infrared absorption spectrum of the C = O stretching vibration. All the C = O double bonds were used for this analysis. The infrared absorption spectrum was calculated in the same way as Ref. [26], as shown in Fig 1A. We determined the resonance wavenumber of this model fibril as being 1680 cm^−1^.

#### 2.2.2 Nonequilibrium molecular dynamics simulation

After determining the resonance wavenumber, we performed nonequilibrium MD simulations of the polyA amyloid fibril in water. As in Ref. [26], a time-varying electric field with the resonance wavenumber was applied to simulate the FEL irradiation. The IR-FEL consists of a series of macro-pulses, and each macro-pulse consists of consecutive micro-pulses. To mimic the FEL irradiation, an electric field was applied as a series of Gaussian-distributed pulses [26, 54] with an interval of 35 ps, as shown in Fig 1B. This pulse corresponds to the micro-pulse in the FEL experiments [14]. Each pulse is expressed as

$$E=E_{0}\exp(-\frac{{\left(t-t_{0}\right)}^{2}}{2\sigma^{2}})\cos(\omega (t-t_{0})),$$

where *E*_0_ is the maximum intensity of the electric field, *t* is time, *t*_0_ is the time at *E* = *E*_0_, *σ* is the standard deviation of the Gaussian distribution, and *ω* is the angular frequency related to the wavenumber *ν* such that *ω* = 2*πcν*, where *c* is the speed of light. The wavenumber *ν* was set to the resonance wavenumber 1680 cm^−1^. *E*_0_ and *σ* were set to 1×10^8^ V/cm and 1 ps, respectively. These parameters of the time-varying electric field were set as the same as those in the previous MD simulation study on Aβ [26] except for the resonance wavenumber. Although the electric field in this MD simulation is larger and irradiated more frequently than those applied for the current wet experiment, we set these parameters to these values to compare the present MD results with the previous MD results and to reduce computation time. Zooming in on the electric field pulse, we can see the oscillations with the wavenumber ν, as shown in Fig 1C. We used the final conformations and velocities in the previous equilibrium MD simulations as the initial conformations and velocities for the nonequilibrium MD simulations. Even though the final simulation box sizes of the six equilibrium MD simulations were slightly different in the order of 0.1 Å, the box size in these nonequilibrium simulations was fixed at 67.978 Å. This value is the average of the final simulation box sizes. We then performed constant-temperature MD simulations at 310 K for 4000 pulses, that is, for 140 ns.

## 3 Results

### 3.1 Reduced aggregation of 13A after FEL irradiation

As for the wet experiment, the following results were obtained. 13A was initially aggregated by incubating the peptide in water (1 mg/ml) at 37°C for 7 days with 140 shakes per minute. The 13A was either subjected to FEL irradiation or left untreated. The wavelength of 6.1 μm was chosen because this wavelength successfully dissociated polyQ aggregates [55].

**Fig 2:**
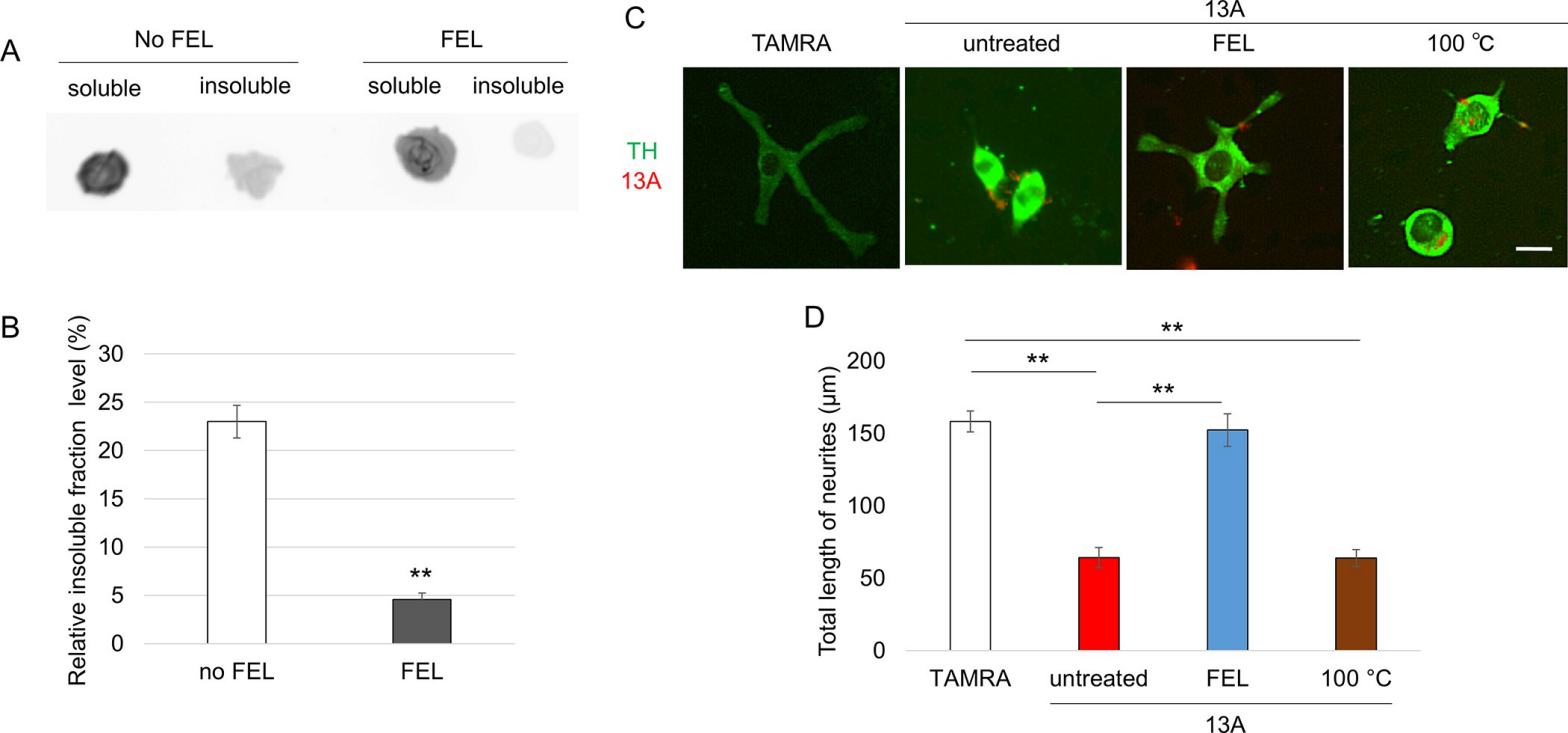
FEL irradiation lowered the extent of 13A aggregation and protected neuron-like cells from 13A-induced cytotoxicity. (A, B) Dot blot analysis. The representative image is shown in panel (A). (B) Relative amounts of 13A in insoluble fractions to amounts in soluble fractions were compared between 13A without and with FEL irradiation (n = 5 each). (C, D) Differentiation of neuron-like PC12 cells after stimulation with NGF. (C) The representative images of PC12 cells with TAMRA (n = 47), non-irradiated 13A (red) (n = 42), irradiated 13A (n = 38) and heated 13A (n = 32). PC12 cells were immunostained with anti-tyrosine hydroxylase (TH) antibody (green). (D) Quantification of the total length of neurites 5 days after the addition of NGF. ANOVA (B) and Kruskal-Wallis (D) were applied. **p < 0.01. Scale bar, 20 μm.

To test if the FEL irradiation weakens the extent of aggregation, we measured the amount of 13A in detergent-soluble and -insoluble fractions. If the 13A is highly aggregated, then 13A can be found in the detergent-insoluble fraction. As shown in Fig 2A and 2B, the amount of 13A in the Triton X-100-insoluble fraction relative to that in the soluble fraction was significantly smaller after FEL irradiation. Thus, FEL irradiation at 6.1 μm reduced the extent of 13A aggregation.

We previously reported that aggregated 13A added in a culture medium was spontaneously taken up by neuron-like PC12 cells and impaired NGF-induced differentiation of the cells [9]. We performed experiments in which the aggregated 13A was either irradiated or left untreated. These 13A peptides were added to PC12 cells, and the cells were cultured for 5 days in the presence of NGF. As a control, TAMRA alone was added to cells. Upon stimulation with NGF, PC12 cells are differentiated and extend neurites. The total length of neurites was shorter in cells treated with non-irradiated 13A than those with TAMRA (Fig 2C and 2D), as seen in the previous literature [9]. Remarkably, the length was not different between cells with TAMRA and those with irradiated 13A (Fig 2C and 2D), suggesting that FEL irradiation to aggregated 13A removed the cytotoxicity of 13A.

FEL irradiation increased the temperature of the entire system by only 1°C [55], but the temperature may have increased more locally and momentarily. Because it was thought such heating might be sufficient to exert the effect, we heated 13A at 100°C for 15 min and introduced the heated 13A into PC12 cells. However, it is not likely that heating alone will dissociate 13A aggregates because the cells with the heated 13A did not show long neurites (Fig 2 and 2D).

### 3.2 Amyloid-fibril dissociation observed by nonequilibrium MD simulation

**Fig 3:**
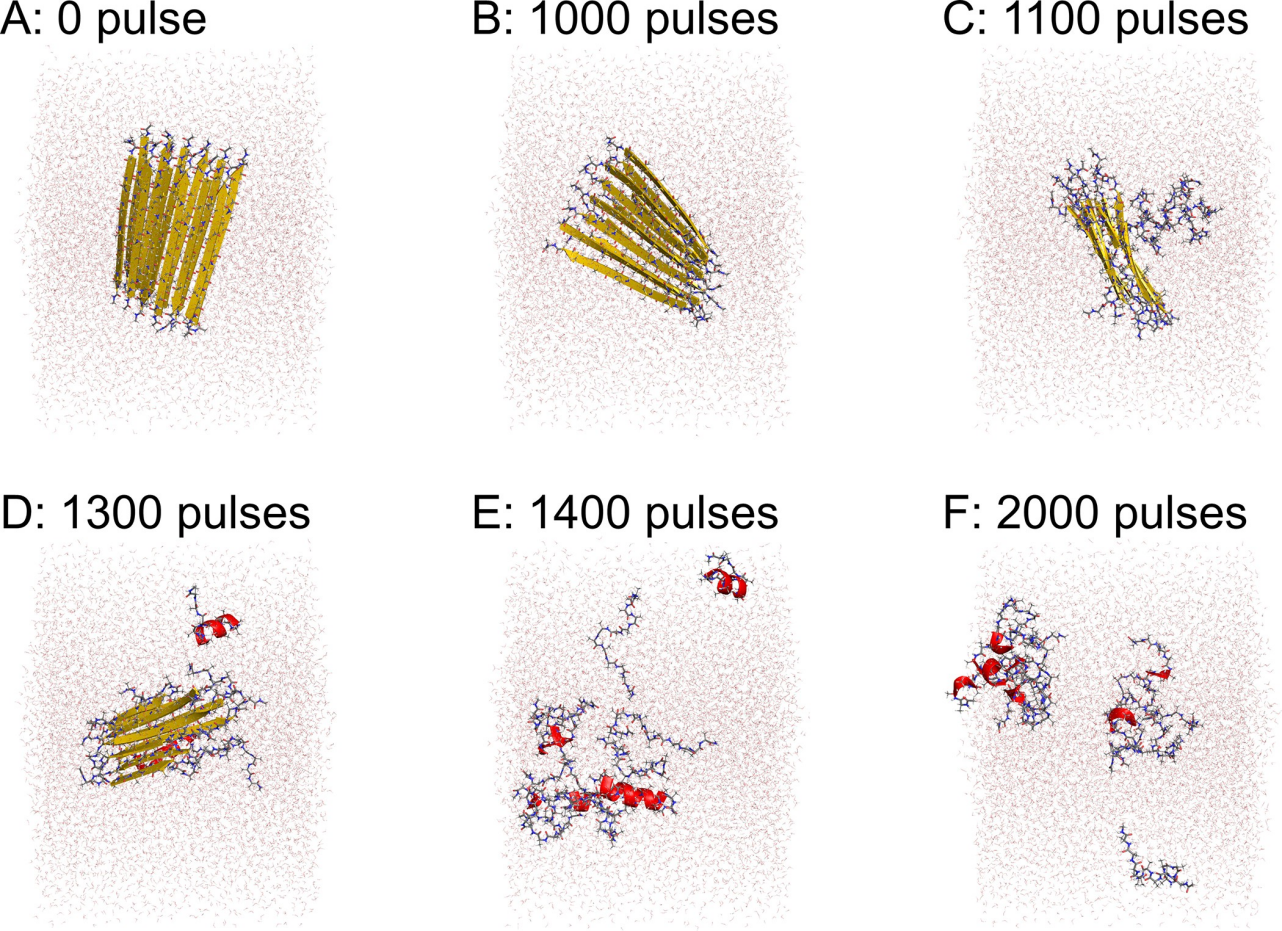
Snapshots of polyA amyloid fibril during a nonequilibrium MD simulation. Snapshots (A) before FEL irradiation, (B) after 1000 pulses, (C) after 1100 pulses, (D) after 1300 pulses, (E) after 1400 pulses, and (F) after 2000 pulses.

The polyA peptides initially had the intermolecular β-sheet structure. This intermolecular β-sheet structure began to break down after 1100 pulses in the trajectory shown in Fig 3 and S2 Movie. About half of the intermolecular β-sheet structure was destroyed after 1300 pulses. Almost all of the intermolecular β-sheet structure was disrupted after 1400 pulses.

**Fig 4:**
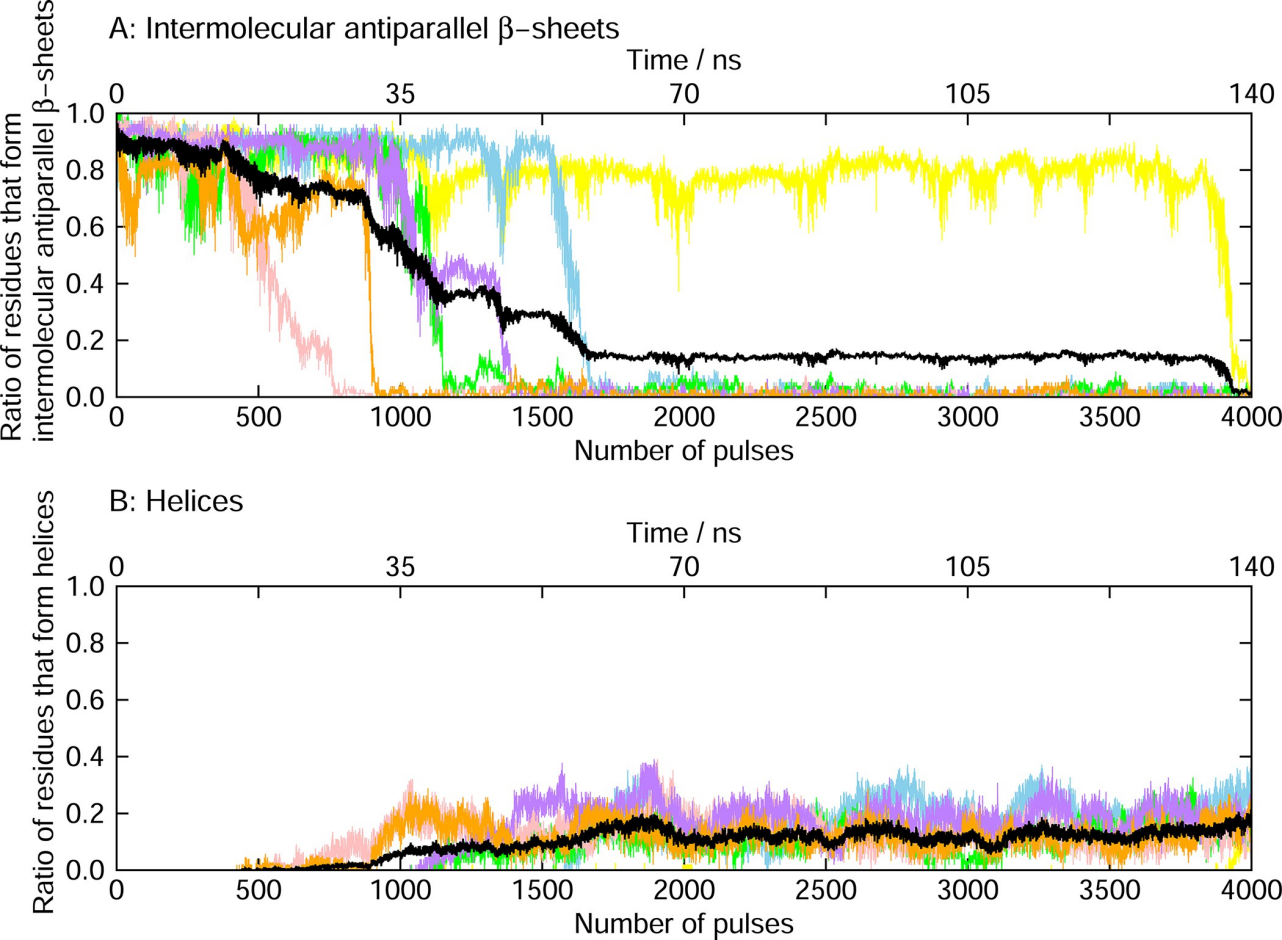
Ratio of the residues that form the secondary structures in the nonequilibrium MD simulations. (A) Intermolecular β-sheets and (B) helices. Different colors mean the results obtained from six different MD simulations. The black line indicates the average of the six MD simulations.

Based on this result, we calculated the ratio of the amino acid residues that formed the intermolecular antiparallel β-sheet and helix structures according to the DSSP criteria [56], as shown in Fig 4. The helix structures here include the α-, 3_10_-, and π-helices [56]. The trajectory shown in Fig 3 and S2 Movie corresponds to the purple lines in Fig 4. Almost all the intermolecular β-sheet structures were destroyed after 4000 pulses in all the MD trajectories. Fig 4B shows that the helix structures increased as the intermolecular β-sheet structure was disrupted. Fig 3 and S2 Movie also show that the helix structures, indicated by red ribbons, increased after the breakdown of the intermolecular β-sheet structure.

**Fig 5:**
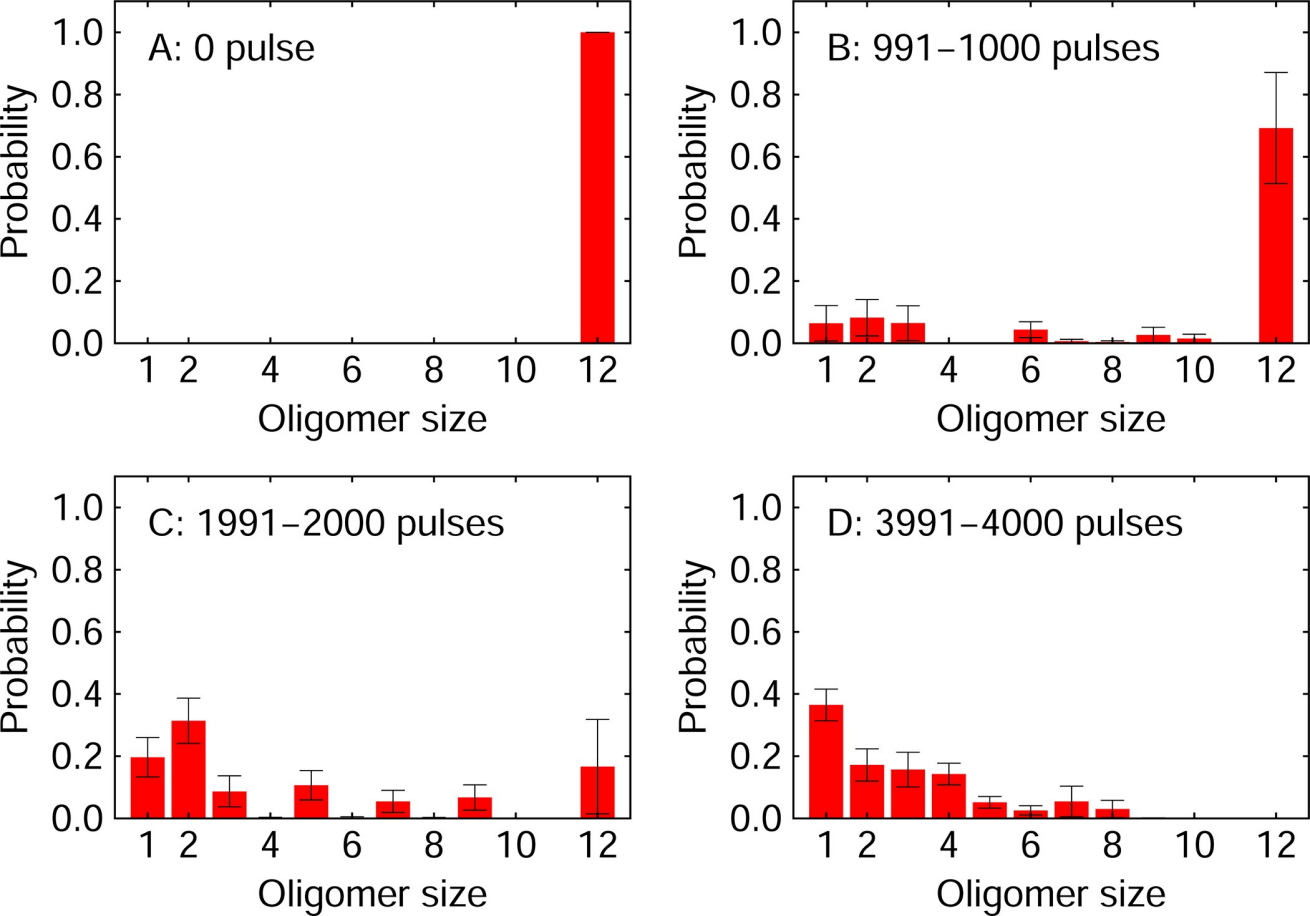
Oligomer size distributions. (A) Oligomer size distribution before the laser irradiation. Averages during (B) 991–1000 pulses, (C) 1991–2000 pulses, and (D) 3991–4000 pulses.

The time variation of the oligomer size distribution during the dissociation of the amyloid fibril was also calculated. When the distance between two heavy atoms (atoms other than hydrogen) in different polyA peptides was within 5 Å, these polyA peptides were regarded to belong to the same oligomer. The results are shown in Fig 5. Before laser irradiation, twelve polyA peptides formed the amyloid fibril. The oligomer size decreased as the amyloid fibril was dissociated by laser irradiation. After 4000 pulses, there were no dodecameric peptides, and most of them were broken down into monomers or small oligomers, such as dimers to tetramers.

**Fig 6:**
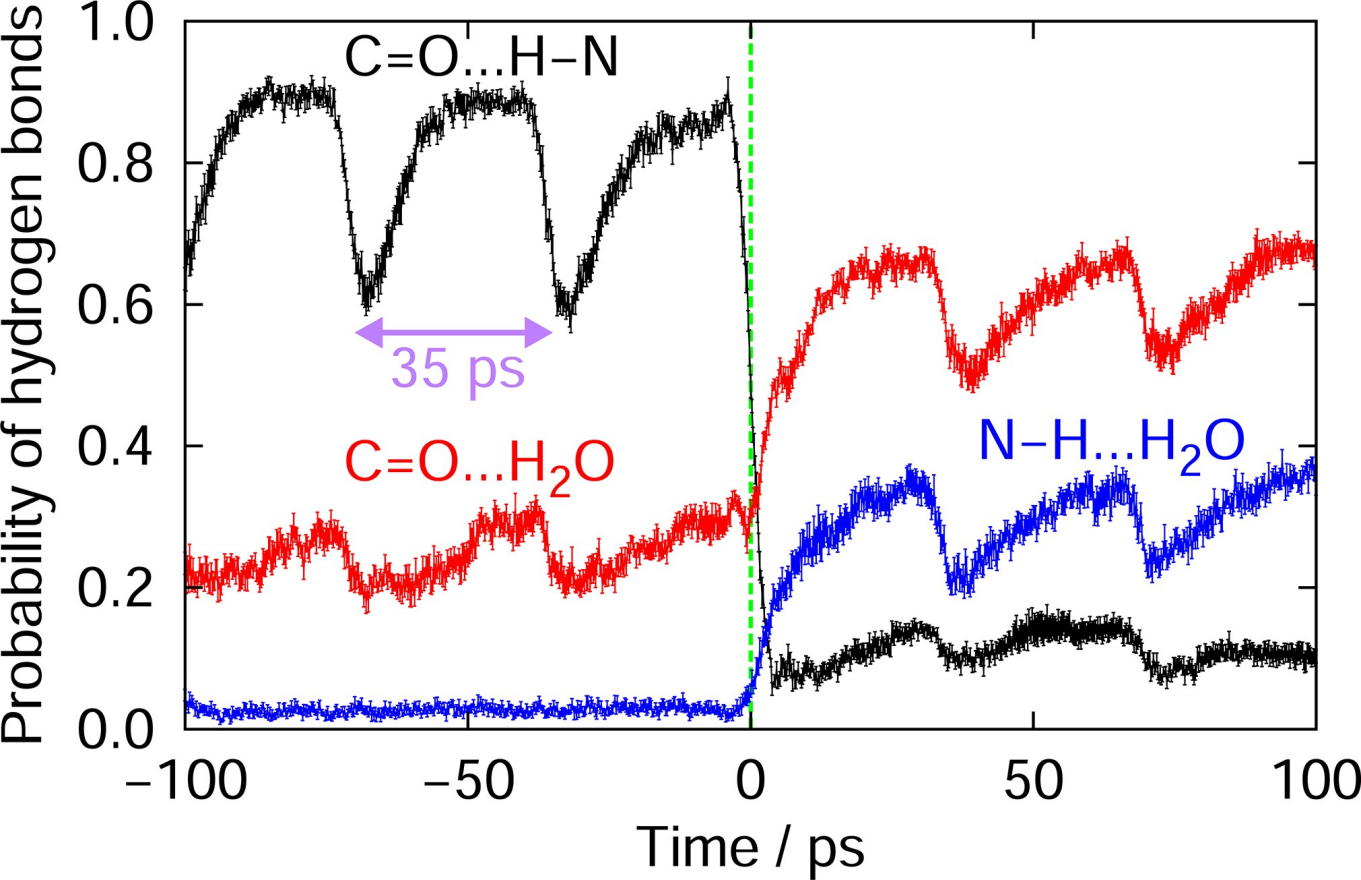
Time series of the probabilities of hydrogen-bond formation. Time series of the averaged probabilities of hydrogen bond formation between C = O and N–H in polyA (black), between C = O and water molecules (red), and between N–H and water molecules (blue). A time of 0 ps means that a set of the hydrogen bonds that form amyloid fibril were broken irreversibly at this time. The averages were then taken for all the C = O and N–H pairs that formed hydrogen bonds in the polyA amyloid fibrils.

Water molecules often play an important role in protein conformational changes [33, 57–64]. To clarify the role of water molecules in the dissociation of the amyloid fibrils, we calculated the probability of hydrogen-bond formation between C = O and N-H, between C = O and water molecules, and between N–H and water molecules in the same way as Ref. [26], as shown in Fig 6. By calculating these quantities, we can find whether a water-molecule penetration breaks the hydrogen bond between C = O and N–H of polyA or whether water molecules enter between the C = O and N–H of polyA after the hydrogen bond between them is broken. Fig 6 indicates that water molecules enter the gaps where the hydrogen bonds between C = O and N–H have been temporally broken by laser irradiation. If a water molecule penetrates and breaks the hydrogen bond between C = O and N–H of polyA, the hydrogen bonds between the polyA peptides and water molecules would increase before the hydrogen bond is broken irreversibly. However, such behavior was not observed. Hydrogen bonds between C = O and water molecules and those between N–H and water molecules increased after the hydrogen bonds between C = O and N–H were broken. We can also see that the hydrogen bonds between C = O and water molecules decreased and recovered repeatedly every 35 ps, the period between the micro-pulses, by the laser pulse irradiation before the C = O and N–H hydrogen bond is broken irreversibly. This time course of the hydrogen-bond formation is the same behavior as that of Aβ amyloid fibril [26].

## 4 Discussion

Both MD simulation and experimental results in this study show that FEL irradiation can dissociate polyA aggregates as well as Aβ aggregates that were already investigated [26, 65]. Since MD simulations can discuss structural changes at the atomic level, we discuss here the dissociation process of polyA aggregates in this simulation. The dissociation process of polyA amyloid fibrils observed here is similar to that of Aβ amyloid fibrils already investigated. However, the dissociation of polyA amyloid fibrils took longer than that of Aβ amyloid fibrils; Aβ amyloid fibrils were dissociated after approximately 1,000 infrared laser pulses [26], whereas polyA amyloid fibrils were dissociated after 4,000 pulses. The average percentage of intermolecular β-sheet formation over the six MD simulations was still half after the 1000 laser pulses, as shown in Fig 4. The reason can be explained as follows. The dissociation mechanism of amyloid fibrils by infrared laser irradiation has already been revealed as follows [26]: Infrared laser irradiation breaks the intermolecular hydrogen bonds between C = O and N–H. These hydrogen bonds are reformed after the laser pulse ends in most cases. However, when water molecules nearby happen to enter the gap between C = O and N–H, the reformation of the hydrogen bonds is inhibited, and the amyloid fibrils are dissociated. PolyA is composed entirely of alanine residues, whereas Aβ consists of both hydrophilic and hydrophobic residues. In general, relatively fewer water molecules exist near hydrophobic residues. During Aβ amyloid fibril dissociation, hydrogen bonds are broken from the hydrophilic residues, Glu22 and Asp23. On the other hand, the amino acid residues in polyA are all the same, alanine, which is a hydrophobic amino acid [26]. Even if an infrared laser pulse irradiation creates a gap between C = O and N–H, the probability of a well-timed water molecule entering the gap is low. PolyA amyloid fibrils are thus less likely to be dissociated than Aβ.

One might think that the difference in the dissociation process of Aβ and polyA amyloid fibrils could simply be due to the difference in size between Aβ and polyA aggregates. However, both model amyloid fibrils used here consist of 12 peptides. Moreover, the number of amino acid residues of polyA (13 residues) is fewer than that of Aβ (42 residues). Despite this, the fact that polyA amyloid fibril required longer irradiation time for dissociation indicates that it is not simply the size effect but that polyA amyloid fibril is more difficult to dissociate than Aβ.

Based on the above discussion, considering a potential application of FEL irradiation to patients, it may be necessary to irradiate polyA disease patients with FEL for a longer period than Alzheimer’s patients, although it depends on the amount and location of the aggregates. In any case, this study shows that polyA aggregates can be dissociated by FEL irradiation in the same way as Aβ aggregates. This suggests that FEL irradiation may have therapeutic potential for patients with polyA disease.

Some readers may wonder why amyloid fibrils of both Aβ and polyA are dissociated by irradiation at 6.1 μm FEL since the resonance wavenumber of the amide I band depends on the three-dimensional structure of the protein. MD simulations show that the resonance wave number of Aβ amyloid fibrils is 1676 cm^-1^, whereas polyA is 1680 cm^-1^. Although the absolute values of these resonance frequencies deviate from the experimental values, the difference between the two fibrils is considered to be almost the same between the MD calculations and experiments. Therefore, the difference between the resonance wavelength of the amyloid fibrils of Aβ and that of polyA is considered to be about 0.2% or 0.01 μm in wavelength. On the other hand, FEL is an ultrashort pulse laser and contains light with a wavelength range of ±3–4% [66, 67]. This is the reason why 6.1 μm FEL can dissociate both Aβ and polyA amyloid fibrils.

Intracellular polyA aggregates should be the primary targets of FEL irradiation. However, it has not been determined if FEL at 6.1 μm deeply penetrates tissues. Interestingly, a general property shared by the protein aggregates is a transfer between cells [68]. Therefore, aggregates are also present in the extracellular space. Extracellular aggregates can be more efficiently irradiated than intracellular aggregates. Thus, when considering the application of FEL to polyA patients, FEL irradiation to extracellular polyA aggregates might be applied.

## Supporting information

S1 Movie Equilibrium molecular dynamics simulation of a polyA amyloid fibril starting from a single-layer β-sheet structure. (AVI)

S2 Movie Dissociation of a polyA amyloid fibril observed by a nonequilibrium molecular dynamics simulation. (AVI)

S1 File Original blot image for Fig 2A. (PDF)

## Abbreviations

polyA: polyalanine
MD: molecular dynamics
FEL: free electron laser
