# Supplementary material for: Dissociation process of polyalanine aggregates by free electron laser irradiation
Source: PLoS One. 2023 Sep 8;18(9):e0291093. doi: 10.1371/journal.pone.0291093 (PMC10491298; doi:10.1371/journal.pone.0291093)
Supplement: S1 File — (PDF) [file pone.0291093.s003.pdf]

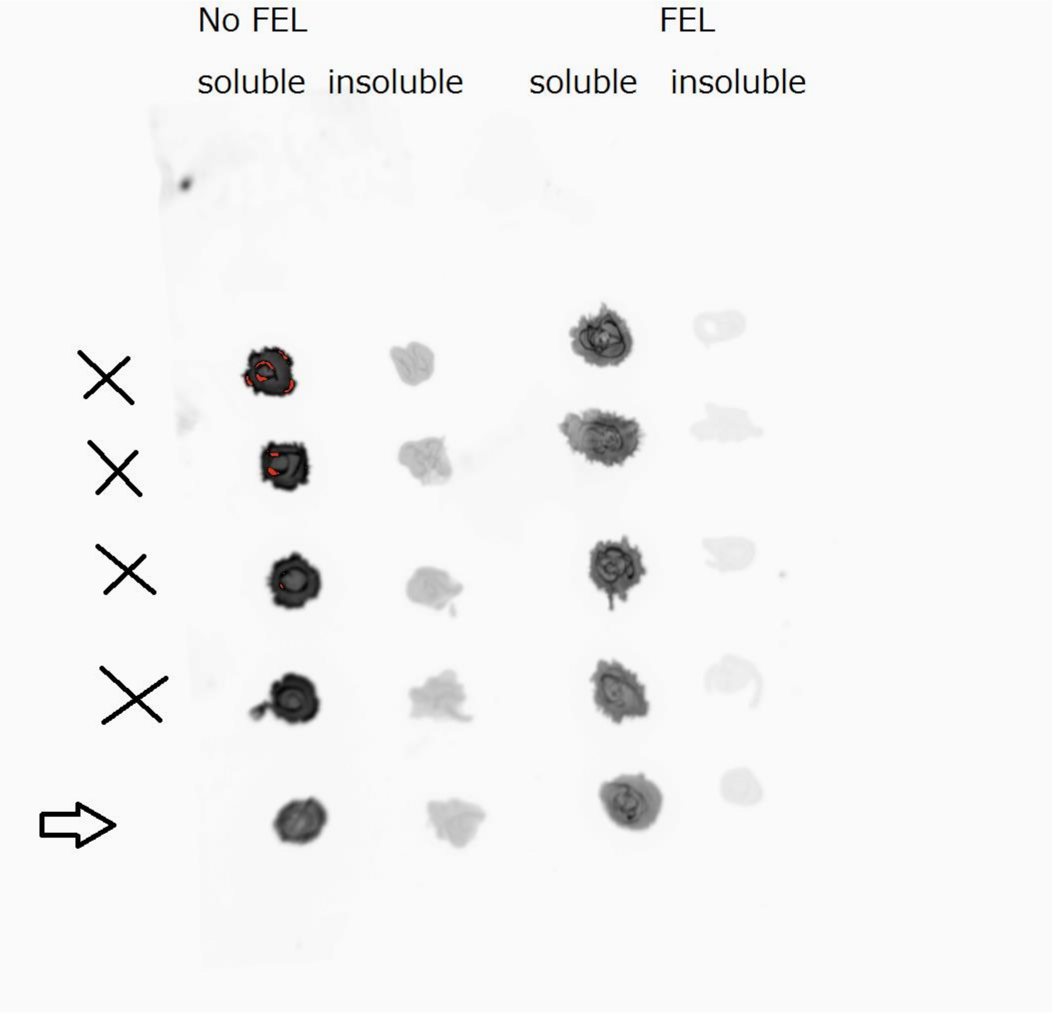

S1_raw_image. Original image for Fig. 2A

One microliters of each fraction of TAMRA-labeled 13A were dropped onto PVDF membrane. The signals on the membrane were obtained using ImageQuant^{TM} LAS 4010 system (exposure type, precision; sensitivity, standard; Iris, F0.85). Multiple pictures were taken by changing the exposure time from 1/100 to 1/4 sec and a best image was selected. The image was saved as a TIFF file.
